# Pooling across cells to normalize single-cell RNA sequencing data with many zero counts

**DOI:** 10.1186/s13059-016-0947-7

**Published:** 2016-04-27

**Authors:** Aaron T. L. Lun, Karsten Bach, John C. Marioni

**Affiliations:** Cancer Research UK Cambridge Institute, University of Cambridge, Li Ka Shing Centre, Robinson Way, CB2 0RE, Cambridge, UK; EMBL European Bioinformatics Institute, Wellcome Genome Campus, Hinxton, CB10 1SD, Cambridge, UK; Wellcome Trust Sanger Institute, Wellcome Genome Campus, Hinxton, CB10 1SA, Cambridge, UK

**Keywords:** Single-cell RNA-seq, Normalization, Differential expression

## Abstract

Normalization of single-cell RNA sequencing data is necessary to eliminate cell-specific biases prior to downstream analyses. However, this is not straightforward for noisy single-cell data where many counts are zero. We present a novel approach where expression values are summed across pools of cells, and the summed values are used for normalization. Pool-based size factors are then deconvolved to yield cell-based factors. Our deconvolution approach outperforms existing methods for accurate normalization of cell-specific biases in simulated data. Similar behavior is observed in real data, where deconvolution improves the relevance of results of downstream analyses.

**Electronic supplementary material:**

The online version of this article (doi:10.1186/s13059-016-0947-7) contains supplementary material, which is available to authorized users.

## Background

Single-cell RNA sequencing (scRNA-seq) is a powerful technique that allows researchers to characterize the gene expression profile of single cells. From each cell, mRNA is isolated and reverse-transcribed into cDNA, which is amplified and subjected to massively parallel sequencing [1]. The sequencing reads are mapped to a reference genome, such that the number of reads mapped to each gene can be used to quantify its expression. Alternatively, transcript molecules can be counted directly using unique molecular identifiers (UMIs) [2]. Count data can be analyzed to identify new cell subtypes and to detect highly variable or differentially expressed (DE) genes between cell subpopulations. This type of single-cell resolution is not possible with bulk RNA sequencing of cellular populations. However, the downside is that the counts often contain high levels of technical noise with many dropouts, i.e., zero or near-zero values. This is due to the presence of low amounts of RNA per cell, which decreases the efficiency with which transcripts can be captured and processed prior to sequencing. Moreover, the capture efficiency often varies from cell to cell, such that counts cannot be directly compared between cells.

Normalization of the scRNA-seq counts is a critical step that corrects for cell-to-cell differences in capture efficiency, sequencing depth, and other technical confounders. This ensures that downstream comparisons of relative expression between cells are valid. Two broad classes of methods for scaling normalization are available: those using spike-in RNA sets and those using the counts from the profiled cellular RNA. In the former, the same quantity of spike-in RNA is added to each cell prior to library preparation [1]. Any difference in the coverage of the spike-in transcripts must be caused by differences in capture efficiency, amplification bias, or sequencing depth between cells. Normalization is then performed by scaling the counts to equalize spike-in coverage between cells. For the methods using cellular counts, the assumption is that most genes are not DE across the sampled cells. Counts are scaled so that there is, on average, no fold-difference in expression between cells for the majority of genes. This is the underlying concept of commonly used methods such as DESeq [3] and trimmed mean of *M* values (TMM) normalization [4]. An even simpler approach involves scaling the counts to remove differences in library sizes between cells, i.e., library size normalization.

The type of normalization that can be used depends on the characteristics of the data set. In some cases, spike-in counts may not be present, which obviously precludes their use in normalization. For example, droplet-based protocols [5, 6] do not allow spike-ins to be easily incorporated. Spike-in normalization also depends on several assumptions [4, 7, 8], the violations of which may compromise performance [9]. Methods based on cellular counts can be applied more generally but have their own deficiencies. Normalization by library size is insufficient when DE genes are present, as composition biases can introduce spurious differences between cells [4]. DESeq or TMM normalization are more robust to DE but rely on the calculation of ratios of counts between cells. This is not straightforward in scRNA-seq data, where the high frequency of dropout events interferes with stable normalization. A large number of zeroes will result in nonsensical size factors from DESeq or undefined *M* values from TMM. One could proceed by removing the offending genes during normalization for each cell, but this may introduce biases if the number of zeroes varies across cells.

Correct normalization of scRNA-seq data is essential as it determines the validity of downstream quantitative analyses. In this article, we describe a deconvolution approach that improves the accuracy of normalization without using spike-ins. Briefly, normalization is performed on pooled counts for multiple cells, where the incidence of problematic zeroes is reduced by summing across cells. The pooled size factors are then deconvolved to infer the size factors for the individual cells. Using a variety of simple simulations, we demonstrate that our approach outperforms the direct application of existing normalization methods for count data with many zeroes. We also show a similar difference in behavior on several real data sets, where the use of different normalization methods affects the final biological conclusions. These results suggest that our approach is a viable alternative to existing methods for general normalization of scRNA-seq data.

## Results and discussion

### Existing normalization methods fail with zero counts

#### The origin of zero counts in scRNA-seq data

The high frequency of zeroes in scRNA-seq data is driven by both biological and technical factors. Gene expression is highly variable across cells due to cell-to-cell heterogeneity and phenomena like transcriptional bursting [7]. Such variability can result in zero counts for lowly expressed genes. It is also technically difficult to process low quantities of input RNA into sequenceable libraries. This results in high dropout rates whereby low-abundance transcripts are not captured during library preparation [10].

At this point, it is important to distinguish between systematic, semi-systematic, and stochastic zeroes. Systematic zeroes refer to genes that are constitutively silent across all cells in the data set, such that the count will be zero for each cell. These are generally not problematic as they contain no information and can be removed prior to normalization. Stochastic zeroes are found in genes that are actively expressed but counts of zero are obtained for some cells due to sampling stochasticity. These genes may contain information about the relative differences between cells, so removing them prior to normalization may introduce biases. We also define semi-systematic zeroes where the gene is silent in a cell subpopulation but is expressed in other cells. This results in zeroes for the silent subpopulation but non-zero counts elsewhere, thus providing information about the differences between subpopulations.

#### A brief description of existing non-spike-in methods

Here, we only consider normalization methods that do not require spike-in data. This is motivated by the desire to obtain a general method that can be applied to all data sets. In particular, we will review three approaches that are commonly used for RNA-seq data: DESeq, TMM, and library size normalization.

DESeq normalization was originally introduced as part of the DESeq package for detecting DE genes [3]. It first constructs an average reference library, in which the count for each gene is defined as the geometric mean of the counts for that gene across all real libraries. Each real library is then normalized against this average. Specifically, for each gene, the ratio of the count in each library to that in the average library is computed. The size factor for each library is defined as the median of this ratio across all genes. The counts in that library are then scaled by the reciprocal of the size factor to eliminate systematic differences in expression between libraries for the majority of (assumed) non-DE genes.

TMM normalization was introduced as part of the edgeR package for DE testing [11]. This method selects one library as a reference and normalizes the remaining libraries against the reference. Specifically, for each remaining library, *M* values (i.e., library size-adjusted log _2_-ratios in expression) are computed against the reference for all genes. The genes with the most extreme *M* values are trimmed away. High- or low-abundance genes are similarly removed. A weighted mean of the remaining *M* values is computed and used to define the normalization factor for each library. Taking the product of the normalization factor and library size for each library (i.e., the effective library size) yields a value that is functionally equivalent to the size factor.

Both DESeq and TMM normalization assume that some minimal proportion of genes are not DE between libraries. For DESeq, the proportion is 50 % of all genes, whereas for TMM, it is 40–70 % depending on the direction of DE [4] (for simplicity, this will be referred to as a non-DE majority for both methods). Consequently, any systematic difference in expression across the majority of genes is treated as bias, which is incorporated into the size/normalization factors and removed upon scaling. If the non-DE assumption does not hold, the computed factors will not be accurate. In addition, both methods perform poorly in the presence of a large number of zeroes. For DESeq normalization, the geometric mean will be equal to zero for genes with a zero count in any library, such that the ratios for that gene become undefined. Moreover, a library with zero counts for a majority of genes will have a size factor of zero, which precludes any sensible scaling. For TMM normalization, *M* values are undefined when the count in either library is zero. In such conditions, both methods require ad hoc workarounds such as the removal of zero counts (this is done automatically by their respective implementations).

Finally, library size normalization is another commonly used approach for normalizing RNA-seq data. This involves scaling the counts such that the library size is the same across libraries, and is the basis for measures of normalized expression like counts or transcripts per million. While simple, this approach is not robust to the presence of DE genes [3, 4]. This means that library size normalization is often inappropriate for real data sets in which DE is likely to occur.

Each of the three methods described above was initially developed for normalization of bulk RNA-seq data. Nonetheless, they have been used extensively in the scRNA-seq literature [12–17]. This motivated us to assess the suitability of these existing methods for normalizing single-cell data.

#### Simulating scRNA-seq data with stochastic zeroes and DE

To test the performance of existing methods, simulated scRNA-seq data were generated with DE genes and a large number of stochastic zeroes. Consider a cell *j* in a subpopulation *s*. This subpopulation may represent cell type or some other biological condition, e.g., drug treatment. For each gene *i* in this cell, the count *Y*_*ij*_ was sampled from a negative binomial (NB) distribution with mean *θ*_*j*_*λ*_*is*_. The *θ*_*j*_ term represents cell-specific biases (e.g., in capture efficiency) that must be normalized out, and is sampled for each cell such that

$$\log_{2}(\theta_{j}) \sim \mathcal{N}(0, 0.25). $$

The *λ*_*is*_ term denotes the expected number of transcripts of gene *i* for cells in subpopulation *s*. It is defined as *λ*_*is*_=*ϕ*_*is*_*λ*_*i*0_ where *ϕ*_*is*_ represents the DE fold change for this gene in this subpopulation, and *λ*_*i*0_ is a gene-specific constant sampled from a gamma distribution with shape and rate parameters set to 2. The NB dispersion is also set for each gene at *φ*_*i*_=0.1. These parameter values were chosen to recapitulate aspects of real data [5] (see Additional file 1: Figure S1). Approximately 40–50 % of all counts are sampled as zero in each simulated library. This is similar to real data where around 60 % of the counts in each cell are stochastic or semi-systematic zeroes.

The simulation design involved 10,000 genes for three subpopulations of 250 cells each. For each subpopulation, a set of *G* genes was randomly chosen. DE was introduced for the chosen genes by setting *ϕ*_*is*_ to some above-unity constant *ϕ*_*s*_ for *p*_*s*_ of the *G* genes (i.e., upregulated) and to 0 for the rest (downregulated, corresponding to semi-systematic zeroes). The value of *ϕ*_*s*_ was set to 5 for all *s*, while *p*_*s*_ was set to 20, 50, and 80 % for the first, second, and third subpopulations, respectively. Sets were also mutually exclusive between subpopulations, i.e., any genes chosen for one subpopulation were not chosen for another subpopulation. This provides each subpopulation with a unique expression signature containing different numbers of DE genes in each direction. For all genes that were not chosen, *ϕ*_*is*_ was set to unity to represent the absence of DE. Simulations were performed for *G*=0 (no DE), 1000 (moderate DE), and 3000 (strong DE). The simulation was also repeated with *G* set to 3000, *p*_*s*_ set to 50 % for all *s*, and *ϕ*_*s*_ set to 2, 5, and 10 for the first, second, and third subpopulations, respectively. This represents an alternative scenario where the number of DE genes in either direction is the same but the magnitude of DE is different between subpopulations. In each simulation scenario, the count for each gene in each cell was sampled from a NB distribution as previously described. The mean of this distribution was defined according to the parameter settings of that scenario (i.e., whether that gene was chosen as DE in the corresponding subpopulation, and if so, the direction and magnitude of DE). A summary of the simulation parameters is provided in Table 1.

**Table 1:** Description of the simulation parameters

Symbol	Description
*θ*_*j*_	Cell-specific bias for cell *j*
*λ*_*is*_	Expected number of transcripts for gene *i* in subpopulation *s*
*ϕ*_*is*_	DE fold change for gene *i* in subpopulation *s*
*λ*_*i*0_	Baseline expectation for the number of transcripts
	(i.e., without DE) for gene *i*
*φ*_*i*_	NB dispersion for gene *i*
*ϕ*_*s*_	DE fold change for all upregulated genes in subpopulation *s*
*G*	Number of unique DE genes in each subpopulation
*p*_*s*_	Proportion of DE genes that are upregulated in subpopulation *s*

*DE* differentially expressed, *NB* negative binomial

DESeq, TMM, and library size normalization were applied to these data. Zero counts were removed prior to or during DESeq and TMM normalization; see “Methods” for more details. The true size factor for each cell is *θ*_*j*_, as it represents the extent of scaling required to remove the cell-specific bias. Estimated size factors from each method were then compared to their true values.

#### Size factor estimates from existing methods are inaccurate

**Fig. 1:**
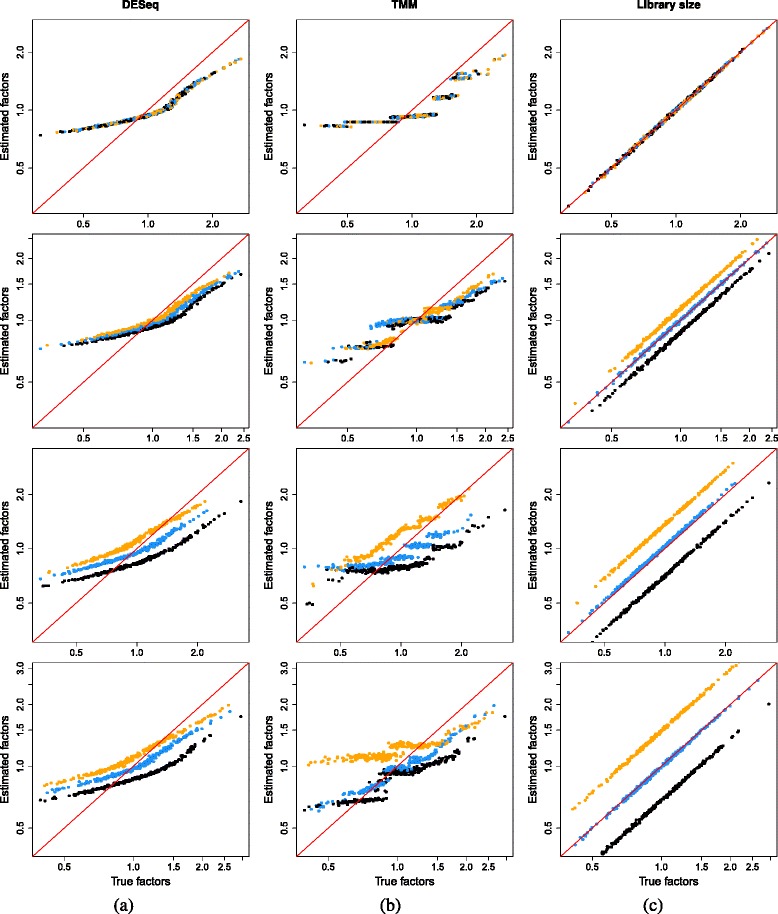
Performance of existing normalization methods on the simulated data with DE genes and stochastic zeroes. The size factor estimates for all cells are plotted against the true values for **a** DESeq, **b** TMM, and **c** library size normalization. Simulations were performed with no DE (*first row*), moderate DE (*second row*), strong DE (*third row*), and varying magnitudes of DE (*fourth row*). Axes are shown on a log-scale. For comparison, each set of size factors was scaled such that the grand mean across cells was the same as that for the true values. The *red line* represents equality between the rescaled estimates and true factors. Cells in the first, second, and third subpopulations are shown in *black*, *blue*, and *orange*, respectively. *DE* differentially expressed, *TMM* trimmed mean of *M* values

**Fig. 2:**
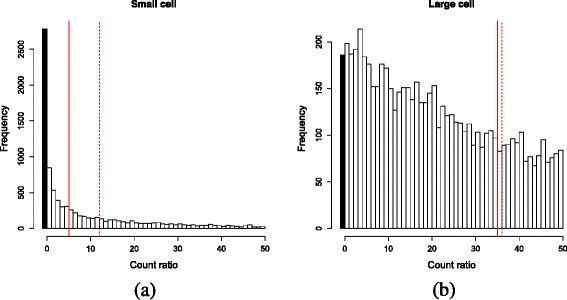
Illustration of the effect of removing stochastic zeroes (*black*) from the distribution of ratios across all genes. Distributions are shown for cells with **a** small and **b** large *θ*_*j*_. The estimated median ratio (*dashed*) is increased beyond the true median (*full*) upon removal of zeroes, which results in overestimation of the size factor for the cell. This effect is more pronounced for cells with small *θ*_*j*_ that have greater numbers of zeroes, compared to cells with large *θ*_*j*_ where the estimated and true medians are more similar

All methods yield size factors that systematically deviate from the true values (Fig. 1). For DESeq and TMM normalization, large size factors are consistently underestimated while small size factors are overestimated. This is a consequence of removing stochastic zeroes prior to normalization. Cells with low *θ*_*j*_ are likely to contain more stochastic zeroes, as the mean of the sampling distribution for the counts is lower. If these zeroes are removed prior to DESeq normalization, the median ratio will be computed from the remaining non-zero counts. This shifts the median upwards and results in overestimation of the size factor (Fig. 2). Similarly, the distribution of *M* values will be shifted towards positive values upon removal of zeroes. This is because stochastic zeroes represent sampled values below some non-zero mean, and would generally correspond to negative *M* values. Their removal increases the (trimmed) mean of *M* values and biases the estimate of the TMM normalization factor. The converse applies to cells with large *θ*_*j*_. Recall that size factors have a relative interpretation across cells, so overestimation of small *θ*_*j*_ will lead to a concomitant underestimation for large *θ*_*j*_.

The presence of DE genes results in a further deterioration in performance of all methods (Fig. 1). The divergence between the true and estimated size factors increases as the number of DE genes increases, consistent with a decrease in the validity of the non-DE assumption required by all methods. To illustrate, consider the strong DE simulation. Across the three subpopulations, there are 9000 genes involved in subpopulation-specific signatures, i.e., 90 % of all genes exhibit DE within this data set. An assumption of a non-DE majority of genes is clearly invalid in this scenario. As a result, the size factors computed from each method will no longer be accurate estimates of *θ*_*j*_, as any systematic difference in expression due to cell-specific biases cannot be distinguished from that due to widespread DE. Normalization inaccuracy is also exacerbated by the removal of semi-systematic zeroes, which distorts the biases between subpopulations in the same way that the removal of stochastic zeroes distorts the biases between cells.

It is worth noting that library size normalization is not affected by stochastic or semi-systematic zeroes. This is because the library size is stably computed by summing across all genes in each cell. Removal of zero counts is not required as they naturally do not contribute to the sum. This results in improved performance in the simple simulation with no DE (Fig. 1c). However, as previously discussed, the use of the library size as a size factor assumes that all genes are non-DE in each cell. Violations of this assumption lead to substantial estimation errors, which can be seen in the results for the simulations involving any DE.

One might attempt to resolve the problem of stochastic zeroes by adding a pseudo-count prior to normalization. This would prevent biases due to unbalanced removal of zeroes between cells. However, direct addition of a pseudo-count squeezes all size factor estimates towards unity (Additional file 1: Figure S2). This is because any ratio of two counts will approach unity when the same pseudo-count is added to both counts. A slightly different approach scales the pseudo-count to match the relative library size of each cell prior to addition. This reduces the bias of the estimates towards unity but is not robust to the presence of DE genes.

### Improving normalization accuracy with deconvolution

#### Overview of the deconvolution strategy

The aim of the deconvolution strategy is to normalize on summed expression values from pools of cells. Summation across cells results in fewer zeroes, which means that the ensuing normalization is less susceptible to the errors observed in the existing methods. While normalization accuracy is improved, the estimated size factors are only relevant to the pools of cells. This is not particularly interesting for downstream analyses, which typically focus on single cells. To obtain relevant estimates, the size factor for each pool is deconvolved into the size factors for its constituent cells. This ensures that cell-specific biases can be properly normalized.

**Fig. 3:**
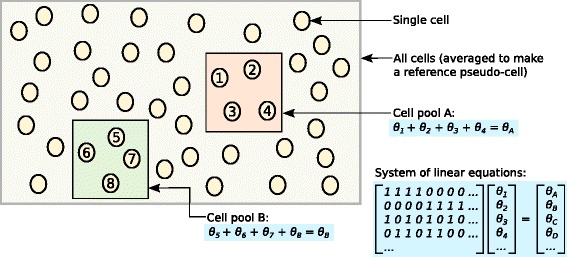
Schematic of the deconvolution method. All cells in the data set are averaged to make a reference pseudo-cell. Expression values for cells in pool A are summed together and normalized against the reference to yield a pool-based size factor *θ*_*A*_. This is equal to the sum of the cell-based factors *θ*_*j*_ for cells *j*=1–4 and can be used to formulate a linear equation. (For simplicity, the *t*_*j*_ term is assumed to be unity here.) Repeating this for multiple pools (e.g., pool B) leads to the construction of a linear system that can be solved to estimate *θ*_*j*_ for each cell *j*

The deconvolution method consists of several key steps:

- Defining a pool of cells
- Summing expression values across all cells in the pool
- Normalizing the cell pool against an average reference, using the summed expression values
- Repeating this for many different pools of cells to construct a linear system
- Deconvolving the pool-based size factors to their cell-based counterparts (Fig. 3)

The following section will describe the implementation of the deconvolution method, as well as its use in conjunction with clustering for optimal performance.

#### Summation and deconvolution with linear equations

Let *Y*_*ij*_ be a random variable representing the count of a non-DE gene *i* in cell *j*, such that *E*(*Y*_*ij*_)=*θ*_*j*_*λ*_*i*0_ where *θ*_*j*_ is the cell-specific bias and *λ*_*i*0_ is the expected transcript count. Define the random variable for the adjusted expression value as $Z_{ij} = Y_{ij}t_{j}^{-1}$, where *t*_*j*_ is a constant adjustment factor for cell *j* (set to the library size, see below) and $E(Z_{ij}) =\theta _{j}\lambda _{i0} t_{j}^{-1}$. Consider a pool *k* consisting of an arbitrary set of cells $\mathcal {S}_{k}$. Define *V*_*ik*_ as the sum of *Z*_*ij*_ across all cells in $\mathcal {S}_{k}$, which has an expectation of

$$ E(V_{ik}) = \lambda_{i0} \sum_{j \in \mathcal{S}_{k}} \theta_{j} t_{j}^{-1}. $$

The observed values of *V*_*ik*_ across all genes constitute an overall expression profile for the pool of cells corresponding to $\mathcal {S}_{k}$. Also define *U*_*i*_ as the mean of *Z*_*ij*_ across all *N* cells in the entire data set, which has an expectation of

$$ E(U_{i}) = \lambda_{i0}N^{-1} \sum_{j \in \mathcal{S}_{0}} \theta_{j} t_{j}^{-1} $$

where $\mathcal {S}_{0}$ refers to the set of all cells in the data set. The observed values of *U*_*i*_ across all genes represent the expression profile for an averaged reference pseudo-cell.

The cell pool *k* is then normalized against this reference pseudo-cell. Define *R*_*ik*_ as the ratio of *V*_*ik*_ to *U*_*i*_ for the non-DE gene *i*. The expectation of *R*_*ik*_ represents the true size factor for the pooled cells in $\mathcal {S}_{k}$, and is written as

(1) $$ {E(R_{ik}) \approx \frac{E(V_{ik})}{E(U_{i})} } = \frac{\sum_{\mathcal{S}_{k}} \theta_{j} t_{j}^{-1}}{ N^{-1} \sum_{\mathcal{S}_{0}} \theta_{j} t_{j}^{-1}} = \frac{\sum_{\mathcal{S}_{k}} \theta_{j} t_{j}^{-1}}{C}   $$

where *C* is a constant that does not depend on the gene, cell, or $\mathcal {S}_{k}$. The approximation assumes that the variance of *U*_*i*_ is small due to the law of large numbers, which is not unreasonable for data sets with hundreds of cells. Denote the realizations of *Y*_*ij*_, *V*_*ik*_, *U*_*i*_, and *R*_*ik*_ as *y*_*ij*_, *v*_*ik*_, *u*_*i*_, and *r*_*ik*_, respectively. The pool-based size factor *E*(*R*_*ik*_) is estimated by taking a robust average (i.e., the median) of *r*_*ik*_ across all genes, under the assumption that most genes are not DE between the pool and the average pseudo-cell. Robustness protects the average against a small number of DE genes with extreme ratios.

Estimates of *E*(*R*_*ik*_) from many pools can be used to obtain an estimate of *θ*_*j*_ for each individual cell. For each pool *k*, a linear equation is set up based on the expression in Eq. 1, by replacing *E*(*R*_*ik*_) with its estimate and treating the $\theta _{j} t_{j}^{-1}$ for $j \in \mathcal {S}_{k}$ as unknown parameters. The constant *C* can be set to unity and ignored, as it does not contribute to the relative differences between size factors. This process is repeated after using different pools of cells to define $\mathcal {S}_{k}$, yielding an overdetermined system of linear equations in which the $\theta _{j} t_{j}^{-1}$ corresponding to each cell is represented at least once. This system can be solved with standard least-squares methods to obtain estimates of $\theta _{j} t_{j}^{-1}$ for all cells (this represents deconvolution of the cell pool factors to the factors for the individual cells, hence the name). Multiplication by *t*_*j*_ for each cell will yield an estimate of *θ*_*j*_.

This approach may seem somewhat circuitous, given that *θ*_*j*_ could be estimated directly from the counts for each individual cell. However, summation reduces the number of stochastic zeroes that cause problems in existing methods. As a result, ratios computed from pooled expression profiles are more accurate. This improvement will propagate back to the estimates of *θ*_*j*_ when the linear system is solved.

#### Constructing the linear system by selecting cell pools

The pool of cells in each $\mathcal {S}_{k}$ is chosen to consist of similar library sizes. Cells in a given cluster are ordered by their total counts and partitioned into two groups, depending on whether the ranking of each cell is odd or even. These cells are arranged in a ring, with odd cells on the left and even cells on the right. Conceptually, one can start at the 12 o’clock position on the ring, for the largest libraries, move clockwise through the even cells with decreasing library size, reach the smallest libraries at 6 o’clock, and then, continue to move clockwise through the odd cells with increasing library size (Additional file 1: Figure S3). For summation, a sliding window is moved cell-by-cell across this ring where each window contains the same number of cells. These cells are used to define a single instance of $\mathcal {S}_{k}$. Thus, each window defines a separate equation in the linear system. The use of a ring means that the window is still defined at the smallest and largest libraries. In contrast, sliding a window across a linear ordering of cells will result in truncated windows at the boundaries.

The pooling of cells with similar library sizes is designed to provide some robustness to estimation errors for small $\theta _{j} t_{j}^{-1}$. For any pool *k*, estimation errors will be present in the pool-based size factor *E*(*R*_*ik*_). This will lead to errors in the estimates of $\theta _{j} t_{j}^{-1}$ when the linear system is solved. A pool comprising cells with larger $\theta _{j} t_{j}^{-1}$ will have larger *E*(*R*_*ik*_) and thus larger estimation errors for *E*(*R*_*ik*_) and each $\theta _{j} t_{j}^{-1}$ (compared to a pool of cells with smaller $\theta _{j} t_{j}^{-1}$). In and of itself, this is not a problem as the errors will be small relative to the large $\theta _{j} t_{j}^{-1}$. However, if a cell with small $\theta _{j} t_{j}^{-1}$ were also present in the pool, the same errors would become large relative to the small true $\theta _{j} t_{j}^{-1}$ for that cell. To mitigate this effect, we assume that the library size is approximately correlated (positively or negatively) with $\theta _{j} t_{j}^{-1}$. For example, upregulation of a subset of genes in a particular cell will drive an increase in its library size and a simultaneous decrease in $\theta _{j} t_{j}^{-1}$ as *t*_*j*_ increases. This results in a negative association between library size and $\theta _{j} t_{j}^{-1}$. Summing adjacent cells in the ring will then yield pools of cells with roughly similar $\theta _{j} t_{j}^{-1}$. This reduces the risk of small errors in *E*(*R*_*ik*_) being transformed into large errors for $\theta _{j} t_{j}^{-1}$. We demonstrate this effect with a simple simulation in Section 1 of Additional file 1, in which the selection of cell pools through the ring arrangement provides a modest improvement in estimation precision compared to the use of random pools.

The total number of equations in the linear system is equal to the number of cells. The $\theta _{j}t_{j}^{-1}$ term for each cell is represented in *w* equations, where *w* denotes the size of the window. By using different values of *w*, additional equations can be added to improve the precision of the estimates. Specifically, values of *w* are set to 20, 40, 60, 80, and 100 by default. These are large enough to obtain stable sums yet small enough to maintain resolution, i.e., by ensuring that cells with very different library sizes are not summed together. This increases the total number of equations in the system and means that each *θ*_*j*_ is represented in 300 equations.

An additional set of equations is added to ensure that the system is solvable. In each additional equation, the $\theta _{j}t_{j}^{-1}$ for each cell is equated to its size factor estimate, obtained by directly normalizing the single-cell counts against the average reference. These equations are assigned very low weights compared to those of the other equations involving summed cells, equal to 10^−6^ (though any small value can be used) and unity, respectively. A weighted least-squares approach is then applied to solve the linear system. In the coefficient matrix of the system, the incorporation of the additional equations ensures that the columns are linearly independent. This ensures that a single solution can be obtained. Due to their low weights, the additional equations will not contribute substantially to the weighted least-squares solution. This means that the estimated values will be driven primarily by the equations for the summed cells. See Section 2 in Additional file 1 for a more detailed discussion of the effect of these additional equations on the system.

#### Obtaining sensible least-squares solutions

The linear system can be solved using standard methods such as those based on the QR decomposition. However, with such methods, it is theoretically possible to obtain negative estimates for *θ*_*j*_. Such values are obviously nonsensical, as counts should not be scaled to yield negative expression values. One situation in which this might occur involves heterogeneous data with a large spread of $\theta _{j}t_{j}^{-1}$ values, such that $\theta _{j}t_{j}^{-1}$ is already close to zero for some cells. Errors in estimation may then be sufficient to push the estimates of *θ*_*j*_ below zero for these cells. Some protection is provided by using linear inverse models in the limSolve package v1.5.5.1 (https://cran.r-project.org/web/packages/limSolve/index.html) [18] to constrain all size factor estimates to non-negative values. This will not provide sensible estimates for the offending cells. These cells will have size factors of zero and should be removed by the user prior to further analysis, as they are likely to represent low-quality libraries. However, the use of limSolve will ensure that the estimates for other cells are not distorted by negative values elsewhere in the system.

The value of *t*_*j*_ is set to the observed library size for each cell. This ensures that the sum *v*_*ik*_ is not dominated by a small number of very large libraries. Information from each cell will be weighted equally when computing the median ratio for each pool, regardless of library size. It also reduces the risk of obtaining negative estimates for small libraries. Such libraries have small *θ*_*j*_ and would be unduly influenced by (relatively) large estimation errors for cells with larger libraries. Note that there are no problems from treating the observed library size as a fixed quantity, as the deconvolution procedure is valid for arbitrary positive values of *t*_*j*_.

Finally, the standard error of the estimated size factors can be obtained after solving the system. This provides a measure of estimation precision and can be used in downstream analyses to account for normalization uncertainty.

#### Clustering to weaken the non-DE assumption

The deconvolution method makes some moderately strong assumptions regarding the nature of DE across the data set. The use of the median is only valid when less than 50 % of genes are DE in any direction in the cell pool compared to the reference pseudo-cell, i.e., less than 50 % of genes can be upregulated and less than 50 % of genes can be downregulated. If more DE genes are present, the median will not represent a robust average across non-DE genes. The above condition generally requires a proportion of genes to be constantly expressed across all cells in the data set, otherwise, all genes could be DE against the average in every pool of cells. It is only guaranteed to be true when that proportion is equal to or greater than 50 % of all genes. Similar requirements are present for DESeq normalization where an average reference is also used.

To reduce the strength of the non-DE assumption, cells can be clustered based on their expression profiles. The deconvolution method is then applied to the cells in each cluster $\mathcal {C}$ separately, where the sets $\mathcal {S}_{k}$ are nested within each $\mathcal {C}$. This normalizes each cell pool of $\mathcal {S}_{k}$ to a cluster-specific reference pseudo-cell for $\mathcal {C}$, yielding a cluster-specific size factor of *f*_*j*_ for cell $j \in \mathcal {C}$ after deconvolution. These cluster-specific size factors must be rescaled before they can be compared between clusters. To do so, the reference pseudo-cells for all clusters are normalized against each other. This is done by selecting a single baseline pseudo-cell against which all other pseudo-cells are normalized. The median ratio $\tau _{\mathcal {C}}$ of the expression values is computed for the pseudo-cell of each cluster against the baseline pseudo-cell (obviously, the cluster chosen as the baseline will have $\tau _{\mathcal {C}}=1$). The final size factor for cell *j* in cluster $\mathcal {C}$ is subsequently defined as $f_{j}\tau _{\mathcal {C}}$.

The use of within-cluster normalization reduces the amount of DE between cells, as all cells in each cluster have similar expression profiles. This avoids inaccurate estimation of the cluster-specific size factors due to violations of the non-DE assumption during deconvolution. Moreover, the pseudo-cells are normalized in pairwise comparisons to a baseline. This further weakens the assumption as a non-DE majority is only required across pairs of pseudo-cells/clusters, rather than across the entire data set. For example, consider five subpopulations where each subpopulation has a unique set of DE genes that is 20 % of all genes. Only 40 % of genes would be DE between any two subpopulations, while 100 % of genes would exhibit some DE across all subpopulations. In this situation, pairwise normalization would clearly be safer as a non-DE majority would be present between each pair.

Any clustering technique can be used to group cells with similar expression profiles prior to deconvolution. We favor hierarchical clustering on rank correlation-based distances as it avoids any circularity between normalization and clustering; see Section 3 in Additional file 1 for more details. All uses of deconvolution in the following text will be performed in conjunction with this clustering approach.

#### Performance of the deconvolution approach on simulated data

**Fig. 4:**
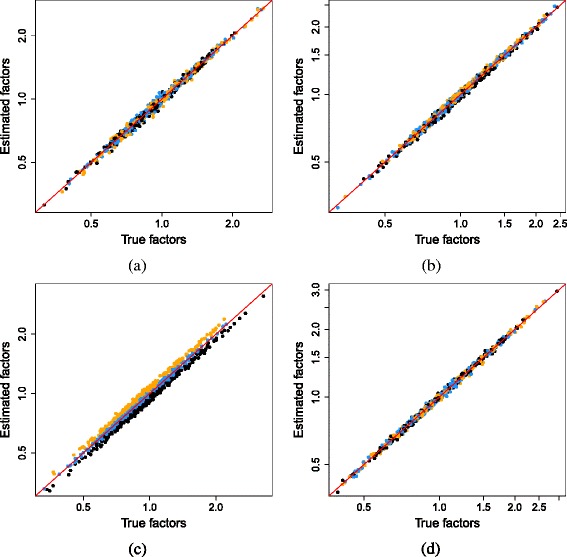
Size factor estimates from the deconvolution method in the simulation with DE genes and stochastic zeroes. These are shown against the true values for scenarios with **a** no DE, **b** moderate DE, **c** strong DE, and **d** varying magnitude of DE. Cells in the first, second, and third subpopulations are shown in *black*, *blue*, and *orange*, respectively. Axes are shown on a log-scale, and the *red line* represents equality with the true factors. *DE* differentially expressed

The deconvolution method provides accurate estimates of the size factor estimates in most simulation scenarios (Fig. 4). This is consistent with the reduced number of stochastic zeroes in the summed counts for each pool of cells. The median ratio for each pool is more accurately computed, which improves the accuracy of the size factor estimates for the individual cells upon deconvolution. Systematic under- or overestimation of the size factors for cells with large or small *θ*_*j*_ is avoided. Some inaccuracy is observed in Fig. 4c, where the non-DE assumption is partially violated by large numbers of DE genes between subpopulations. However, the deconvolution method is still more accurate than the existing methods in the third row of Fig. 1. Unlike DESeq or TMM normalization, the estimates here are proportional to the true values within each subpopulation, and the deviation from the diagonal is smaller than that for library size normalization.

The simulations described above focus on low-coverage data where zeroes are expected to be prevalent. To examine the performance of the methods at higher coverage, we repeated the simulations with different parameters to obtain larger counts (Section 4, Additional file 1). This yielded similar results to Figs. 1 and 4. DESeq and TMM normalization were still inaccurate in the presence of zeroes, while library size normalization failed in the presence of DE genes (Additional file 1: Figure S4). Deconvolution remained the most accurate method in all scenarios (Additional file 1: Figure S5). This suggests that the advantages provided by deconvolution are relevant in scRNA-seq experiments with greater sequencing depth.

Deconvolution can also be assessed in terms of its computational complexity. In the worst-case scenario, the time required by the method will scale in a cubic manner with respect to the number of cells. However, this can be substantially mitigated by clustering to break up the linear system. This is described in more detail in Section 5 of Additional file 1, along with empirical timings in Additional file 1: Figure S6.

### Differences in normalization are recapitulated in real data

#### Overview of the data sets

We also examined the behavior of the deconvolution method in real scRNA-seq data. The first data set involves over 3000 cells from the somatosensory cortex and hippocampal region of the mouse brain [19]. This includes a number of different cell types such as oligodendrocytes, microglia, and various neuronal subtypes. The second data set was generated using the inDrop protocol on mouse embryonic stem cells, before and after withdrawal of leukemia inhibitory factor (LIF) [5].

#### Deconvolution yields a wider spread of size factors

**Fig. 5:**
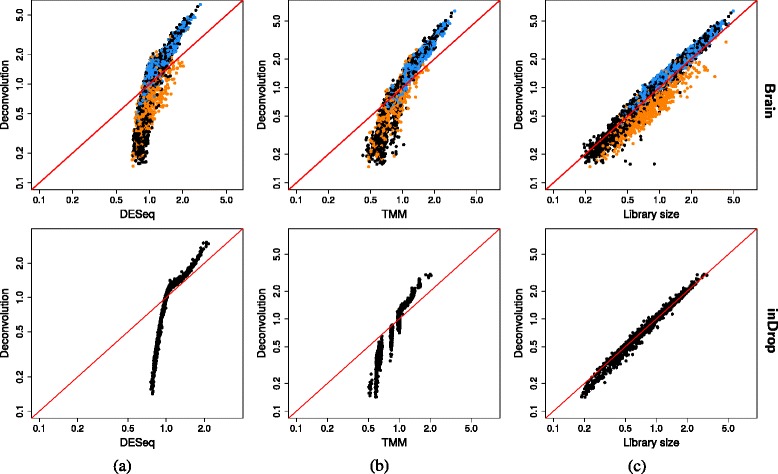
Comparisons between the estimated size factors. Those from the deconvolution method are compared to those from **a** DESeq, **b** TMM, and **c** library size normalization. This is shown for the brain (*top*) and inDrop data sets (*bottom*). Axes are on a log-scale, and the *red line* represents equality between the two sets of factors. All sets of factors were centered to a median of unity prior to comparison. For the brain data, cells classified by Zeisel et al. as oligodendrocytes or pyramidal CA1 cells are shown here in *orange* and *blue*, respectively. *TMM* trimmed mean of *M* values

Each normalization method was applied to the counts for cells in both data sets. In both cases, deconvolution yields a wider range of size factor estimates compared to DESeq and TMM normalization (Fig. 5). This is consistent with the simulation results where the stochastic zeroes cause the estimates to be biased towards unity for these two methods. Indeed, approximately 60 % of counts are equal to zero in each cell for both data sets, even after the removal of low-abundance genes. This indicates that the presence of stochastic zeroes is an intrinsic property of high-throughput scRNA-seq data that cannot simply be ignored. Recall that the deconvolution method reduces the number of zero counts by summing across cells. This avoids bias towards unity in the simulations and increases the range of the estimates in the brain and inDrop data.

Deconvolution also yields different size factors from library size normalization in the brain data (Fig. 5c). Specifically, the majority of oligodendrocytes have size factor estimates from library size normalization that are larger than those from deconvolution, while the opposite is true for the majority of pyramidal CA1 cells. This is attributable to the likely presence of DE genes between the different cell types. For example, any upregulation of genes in oligodendrocytes will increase the size factor estimates from library size normalization for those cells by increasing their library sizes. In contrast, deconvolution uses a median-based approach that is robust to extreme ratios caused by DE. The two methods are more similar for the inDrop data where less DE is expected between cells from the same, theoretically homogeneous, population.

#### Different normalization schemes change the DE profile

To gauge the impact of employing different normalization methods, edgeR was used to perform a DE analysis on both data sets with the different size factor estimates. For the brain data set, the count data were subsetted to contain only cells classified as pyramidal CA1 or oligodendrocytes [19]. DE genes were then detected between cell types at a false discovery rate (FDR) of 5 %. This process was repeated for the inDrop data to test for DE after LIF withdrawal.

The most noticeable difference between methods is observed in the number of DE genes and the signs of their log-fold changes for the brain data set (Table 2). Deconvolution yields a more balanced set of DE genes than DESeq, TMM, or library size normalization, with smaller differences between the numbers of up- and downregulated genes. The set of DE genes detected in either direction also tends to be a superset or subset of that detected by the existing methods. This is consistent with a difference in global scaling of the average expression values for each cell type, such that the DE log-fold changes from deconvolution are shifted in one direction relative to those from the existing methods. For example, consider the differences in the numbers of DE genes between deconvolution and library size normalization. The increase in the number of oligodendrocyte-upregulated genes (i.e., down relative to pyramidal cells) for deconvolution is consistent with the smaller size factors for these cells in Fig. 5c relative to the factors from library size normalization. This is because any increase in gene expression in oligodendrocytes is incorporated into the library size and is weakened upon library size normalization.

**Table 2:** Number of DE genes detected by edgeR at a FDR of 5 % in each data set, using the size factor estimates from different methods for normalization

Method	Brain				inDrop		
	Total	Down	Up		Total	Down	Up
DEseq	5073	894	4179		497	298	199
TMM	4489	1051	3438		462	239	223
Library size	4258	1176	3082		492	199	293
Deconvolution	3632	1706	1926		489	212	277
shared with DESeq	2820	894	1926		411	212	199
shared with TMM	2977	1051	1926		435	212	223
shared with library size	3102	1176	1926		475	198	277

This is also separated into the number of up- and downregulated genes for each data set. Upregulation refers to increased expression in pyramidal CA1 cells over oligodendrocytes in the brain data, and to increased expression after LIF withdrawal in the inDrop data. The number of DE genes shared between analyses using deconvolution and each other method is also shown

*DE* differentially expressed, *FDR* false discovery rate, *LIF* leukemia inhibitory factor, *TMM* trimmed mean of *M* values

In summary, over a thousand genes are lost or gained in the brain data set when deconvolution is used instead of the other methods. This is likely to have some effect on the biological conclusions due to changes in detection power and FDR control, especially if log-fold changes are distorted by incomplete removal of cell-specific biases with existing methods. To demonstrate, a gene set enrichment analysis was conducted with topGO [20] on the unique DE genes detected by either library size normalization or deconvolution. Genes unique to deconvolution were associated with expected biological processes for oligodendrocytes, e.g., amide and lipid metabolism, cell adhesion and membrane organization (Additional file 2). In contrast, genes unique to library size normalization were associated with more varied processes including meiosis and spermatogenesis (Additional file 3). These results suggest that the genes unique to deconvolution are more biologically relevant than those unique to library size normalization. This implies that any conclusions that are taken from the DE analysis will be more valid when deconvolution is used.

The different normalization strategies also affect the ranking of the top set of genes in the brain data. Of the top genes with the lowest *p* values, around 20–50 % are changed when deconvolution is used instead of the existing methods (Additional file 1: Table S1). This is due to a global shift in the log-fold changes between methods, which alters the relative significance of up- and downregulated genes. Modest changes were also observed in the ranking of highly variable genes detected by the distance-to-median approach [13]. Approximately 10 to 30–40 % of the most variable genes were altered if deconvolution was used instead of the existing methods in either data set (Additional file 1: Table S2). Rankings are important as the genes contributing to the biological differences between cells or conditions are expected to have strong variability and DE, respectively. Thus, the top-ranked genes are often prioritized for further investigation. Changes to the rank indicate that the choice of normalization strategy will affect the biological conclusions of the study.

Smaller differences are observed for the inDrop data set where fewer DE genes are present (Table 2, Additional file 1: Table S1). Here, the similar performances of deconvolution and library size normalization are attributable to their mutual robustness to stochastic zeroes and, for the latter, a relative lack of DE within a cell type. This suggests that library size normalization may be satisfactory for homogeneous data sets.

#### Deconvolution increases normalization accuracy on real data

We use a simple offset/covariate approach based on generalized linear models (GLMs) to assess the accuracy of deconvolution compared to each existing method. Briefly, we subset each real data set to contain only cells from one group, e.g., oligodendrocytes in the Zeisel et al. data set. We assume that no DE is present within the group, such that the only differences in the mean counts between cells are due to cell-specific biases. We fit a GLM to these counts, using the log-size factors from deconvolution as offsets and the log-size factors from an existing method as a covariate in the model. If deconvolution is accurately estimating the cell-specific biases, the offsets will recapitulate all of the differences in means between cells in the group. Additional fitting from the covariate term is unnecessary, such that the corresponding coefficient will be zero. This means that, if we were to test against the null hypothesis that the coefficient of the covariate term was equal to zero, we should observe few or no rejections. On the other hand, if deconvolution is not accurate, the offsets alone will fail to recapitulate the differences in means. To capture the remaining differences, the estimated coefficient for the covariate term will become non-zero (assuming the normalization bias and covariate are correlated) such that more rejections will be observed when we test against the null hypothesis.

We perform this process twice: once as described above, and again after switching the size factors, i.e., using size factors from the existing method as the offsets and those from deconvolution as the covariates. We assess the relative accuracy of deconvolution based on the number of DE genes (i.e., with strong evidence against the null hypothesis of a coefficient of zero for the covariate term) in the original and switched GLM fits. If deconvolution is more accurate than the existing method, there should be fewer DE genes when the deconvolution size factors are used as offsets, compared to when they are used in the switched fit as covariates. This is observed for all groups in all tested data sets (Additional file 1: Figure S7), consistent with increased accuracy in the simulations. A more detailed explanation of this evaluation framework is provided in Section 6 of Additional file 1.

## Conclusions

Here, we have presented a normalization strategy for scRNA-seq data based on summation of expression values and deconvolution of pooled size factors. This approach provides improved performance for size factor estimation compared to existing methods on simulated data. In particular, it avoids estimation inaccuracy in the presence of stochastic zeroes and is robust to DE in the data set. Similar differences in the size factors across methods were also observed in analyses of real data, where the use of different size factor sets resulted in changes to the number and identity of detected DE genes. This indicates that the choice of normalization method has a substantial impact on the results of downstream analyses. Any increase in accuracy from our deconvolution approach is likely to have a beneficial effect on the validity of the biological conclusions.

## Methods

### Implementation of existing normalization methods

For DESeq normalization, the geometric mean for each gene was computed after removing all zeroes. This is necessary to avoid a situation where a majority of genes have geometric means of zero, such that the majority of ratios to the geometric mean would be undefined. Size factors were then computed using the estimateSizeFactorsForMatrix function in DESeq2 v1.10.1 [21]. In this function, ratios of zero were automatically removed prior to calculation of the median in each library, to avoid obtaining a size factor equal to zero. For TMM normalization, the calcNormFactors function in the edgeR package v3.12.0 [11] was used with default settings. All undefined *M* values were automatically removed prior to trimming and calculation of the normalization factor for each library. The corresponding size factor was defined as the effective library size, i.e., the product of the library size and the normalization factor for each library. For library size normalization, the total library size was used directly as the size factor for each cell.

### Implementation of the deconvolution method

We have implemented our deconvolution approach as a R function, with C++ extensions for fast construction of the linear system. It is publicly available as the computeSumFactors function in the scran package on Bioconductor (http://bioconductor.org/packages/scran) under the GNU General Public Licence v3.

### Obtaining the real scRNA-seq data

Libraries in the brain data set were prepared for over 3000 single cells using the Fluidigm C1 system [19]. Gene expression was quantified for each cell by counting UMIs after sequencing. Counts for all cells were obtained from http://linnarssonlab.org/cortex. For the inDrop data set, libraries were prepared for over 10,000 cells and quantification was performed with UMIs [5]. Counts were obtained from the NCBI Gene Expression Omnibus with the accession GSE65525.

For both data sets, low-abundance genes were defined as those with an average count below 0.2 across all cells. These were considered to be systematic zeroes (with some non-zero counts due to residual transcription, mapping errors, etc.) and removed prior to further analysis. For the brain data set, spike-in transcripts were removed. This ensures that normalization is only performed using the counts for the cellular genes. For the inDrop data set, counts were only used for cells before withdrawal of LIF and those 7 days after withdrawal. This resulted in a final set consisting of approximately 1700 cells. Cells in the intervening time points were not considered as the library sizes were too small. The average total count across all genes was around 5000 for those cells, compared to 27,000 for the cells in the final set and a median total of around 19,000 for the brain data set.

### Downstream analyses on real data

A DE analysis was performed on each data set using the statistical methods in edgeR. Size factors from each method were used as the effective library sizes. The estimateDisp function was used to estimate a gene-specific NB dispersion for each gene [22] without any empirical Bayes shrinkage. A GLM was fitted for each gene using a one-way layout with the two groups of interest [23]. For the brain data set, the groups are defined according to the cell type, while for the inDrop data set, the groups refer to the time before/after LIF withdrawal. The glmTreat function was used to detect genes with a DE log-fold change significantly greater than 1 between the groups [24]. DE genes were defined at a FDR threshold of 5 % after applying the Benjamini–Hochberg correction to the *p* values.

A gene-ontology (GO) analysis was conducted to characterize the general function of the unique DE genes from deconvolution in the brain data set. Unique DE genes were defined as those detected after deconvolution at a FDR of 5 % that were not detected by the most similar existing method, i.e., library size normalization. The topGO method [20] was then applied to identify GO terms that were enriched within this unique set. GO terms were only considered if they referred to a biological process and contained at least ten genes. The significance of enrichment of GO terms in the unique set was determined using Fisher’s exact test. Top-ranking GO terms were identified based on their enrichment *p* values. This was repeated using the unique DE genes from library size normalization that were not detected after deconvolution.

Highly variable genes in each data set were characterized using the distance-to-median approach [13]. For each normalization method, normalized expression values were computed by dividing each count by the corresponding size factor. The mean and coefficient of variation of these expression values were computed across all cells. The variability of gene expression was quantified by computing the distance-to-median, i.e., the difference between the squared coefficient of variation for each gene to a running median across all genes of similar abundance [13]. The most variable genes were identified as those with the largest distance-to-median statistics.

## Availability of data and materials

All data sets can be downloaded as described in the “Methods” section “Obtaining the real scRNA-seq data”. All R packages can be installed from the Bioconductor repositories (http://bioconductor.org/install). All simulation and analysis code used in this study are available on GitHub (https://github.com/MarioniLab/Deconvolution2016).

## Ethics approval

Not applicable.

## Additional file

Additional file 1 Supplementary materials. This file contains a justification of the choice of pooling strategy (Section 1), a discussion of how to resolve linear dependencies (Section 2), details on the clustering algorithm (Section 3), a description of the high-coverage simulations (Section 4), an outline of the computational complexity of deconvolution (Section 5), and a description of the offset/covariate method used to assess normalization accuracy on real data (Section 6). It also contains Supplementary Figures S1–S7 and Supplementary Tables S1 and S2. (560 KB PDF)

Additional file 2 Enriched GO terms for deconvolution. This file is in a tab-separated format and contains the top 200 GO terms that were enriched in the set of DE genes unique to deconvolution. The identifier and name of each term is shown along with the total number of genes associated with the term, the number of associated genes that are also DE, the expected number under the null hypothesis, and the Fisher *p* value. (13 KB PDF)

Additional file 3 Enriched GO terms for library size normalization. This file is in a tab-separated format and contains the top 200 GO terms that were enriched in the set of DE genes unique to library size normalization. The fields are the same as described for Additional file 2. (13 KB PDF)
