# Supplementary material for: Pooling across cells to normalize single-cell RNA sequencing data with many zero counts
Source: Genome Biol. 2016 Apr 27;17:75. doi: 10.1186/s13059-016-0947-7 (PMC4848819; doi:10.1186/s13059-016-0947-7)
Supplement: Supplementary file 1 — Supplementary materials. This file contains a justification of the choice of pooling strategy (Section 1), a discussion of how to resolve linear dependencies (Section 2), details on the clustering algorithm (Section 3), a description of the high-coverage simulations (Section 4), an outline of the computational complexity of deconvolution (Section 5), and a description of the offset/covariate method used to assess normalization accuracy on real data (Section 6). It also contains Supplementary Figures S1–S7 and Supplementary Tables S1 and S2. (560 KB PDF) [file 13059_2016_947_MOESM1_ESM.pdf]

Pooling across cells to normalize single-cell RNA sequencing data with many zero counts

Supplementary Materials

by

Aaron T. L. Lun[1], Karsten Bach[2] and John C. Marioni[1,2,3]

[1]Cancer Research UK Cambridge Institute, University of Cambridge, Li Ka Shing Centre, Robinson Way, Cambridge CB2 0RE, United Kingdom

[2]EMBL European Bioinformatics Institute, Wellcome Genome Campus, Hinxton, Cambridge CB10 1SD, United Kingdom

[3]Wellcome Trust Sanger Institute, Wellcome Genome Campus, Hinxton, Cambridge CB10 1SA, United Kingdom

April 11, 2016

1 Justifying the choice of pooling strategy

The performance of a ring arrangement and sliding window can be compared to selection of random pools of cells. Count data was simulated as described in the main text, with some modifications – specifically, true values of θ_j for all cells were sampled from a Uniform(0.1, 1) distribution instead, to provide a larger range of size factors; counts were only generated for a single subpopulation of 200 cells; and no DE genes were introduced. To estimate the size factors, deconvolution was performed using pools of 20 cells, assembled either with the ring arrangement in Figure S3 or after randomly permuting the order of cells in the ring. (For demonstration purposes, only one pool size was used to construct the linear system. This ensures that the estimation errors are large enough to reliably observe any changes in precision.) Precision was quantified as the median absolute deviation (MAD) of the log-fold differences between the estimated and true θ_j. A completely precise method should have a MAD of zero, as the estimated and true θ_j would be proportionally identical for all j. In this simulation, using the ring arrangement yields a MAD of 0.051 while using random pools yields a MAD of 0.079 (standard errors of 0.002 and 0.003, respectively, across 10 simulation iterations). This suggests that the ring arrangement provides a modest improvement in estimation precision.

2 Resolving linear dependencies in the constructed system

Consider the application of the deconvolution method on a data set with four cells using a sliding window of size 2. Assuming cells $j = 1$ to 4 were placed consecutively on the ring, this would yield the linear system

$$\begin{bmatrix} 1 & 1 & 0 & 0 \\ 0 & 1 & 1 & 0 \\ 0 & 0 & 1 & 1 \\ 1 & 0 & 0 & 1 \end{bmatrix} \begin{bmatrix} \theta_1 \\ \theta_2 \\ \theta_3 \\ \theta_4 \end{bmatrix} = \begin{bmatrix} \theta_A \\ \theta_B \\ \theta_C \\ \theta_D \end{bmatrix}$$

for pool-based size factors θ_A to θ_D. We set all t_j to unity for simplicity, though the reasoning below applies when solving for both θ_j and $\theta_j t_j^{-1}$. Assume that the pool-based factors are estimated accurately and precisely, such that the minimum value of the residual sum of squares for this system can be obtained near the set of true values for the cell-based factors. This system has no unique solution – for the true factors $(\theta_1, \theta_2, \theta_3, \theta_4)^T$, an equally good fit can be obtained with $(\theta_1 + x, \theta_2 - x, \theta_3 + x, \theta_4 - x)^T$ for any real x.

The addition of equations relating each size factor to its direct estimate $\tilde{\theta}_j$ ensures identifiability, as a value of x will be chosen that minimizes the residual sum of squares of $(\theta_1 + x, \theta_2 - x, \theta_3 + x, \theta_4 - x)^T$ from the direct estimates. In this example, the residual sum of squares for the possible solutions is written as

$$\sum_{j \in J_1} (\theta_j + x - \tilde{\theta}_j)^2 + \sum_{j \in J_2} (\theta_j - x - \tilde{\theta}_j)^2 = 4x^2 + 2x \left[\sum_{j \in J_1} (\theta_j - \tilde{\theta}_j) - \sum_{j \in J_2} (\theta_j - \tilde{\theta}_j) \right] + \sum_{j \in \{J_1, J_2\}} (\theta_j - \tilde{\theta}_j)^2$$

where $J_1 = \{1, 3\}$ and $J_2 = \{2, 4\}$. For these 4 cells, the above expression is minimized in terms of x at

$$x = -\frac{1}{4} \left[\sum_{j \in J_1} (\theta_j - \tilde{\theta}_j) - \sum_{j \in J_2} (\theta_j - \tilde{\theta}_j) \right] .$$

This is true regardless of the relative weighting applied to the set of additional equations. The direct estimator of each size factor is also unbiased as stochastic zeroes are not removed, i.e., $E(\tilde{\Theta}_j) = \theta_j$ for $\tilde{\theta}_j \sim \tilde{\Theta}_j$.

As the number of cells in the system increases, the sizes of J_1 and J_2 will increase. For example, deconvolution is typically applied in data sets involving at least 200 cells and a sliding window of size 20, such that both J_1 and J_2 will consist of at least 10 cells. With more cells, the sum of the errors $\theta_j - \tilde{\theta}_j$ will approach the sum of their expected values, i.e., zero. This means that the minimum residual sum of squares will be obtained at $x = 0$, i.e., solving the linear system will yield the true values of the size factors. Thus, the additional equations will not affect accurate estimation of the size factors in the deconvolution method.

3 Implementation details of the clustering approach

Let ρ_{xy} denote Spearman's rank correlation coefficient between the counts of cells x and y. We define the distance between these cells as $1-\rho_{xy}$. In this manner, a distance matrix is constructed between all pairs of cells. Hierarchical clustering is performed on this matrix using the hclust function with Ward's clustering criterion. Clusters of cells are defined using a dynamic tree cut from the dynamicTreeCut package v1.62 (https://cran.r-project.org/web/packages/dynamicTreeCut/index.html). This ensures that each cluster contains a minimum number of cells (200 by default) required for stable deconvolution. Correlation-based clustering is appealing as it is insensitive to global scaling of the expression values in each cell. Prior normalization is not required, which avoids a circular dependence between normalization and clustering. Alternatively, one can use known aspects of the data set as clusters, e.g., groupings, batches. Empirical clustering may not be required if such information is available, which reduces computational work and reduces the potential for errors.

By default, the baseline pseudo-cell is chosen from the cluster where the mean library size per cell is equal to the median of the mean library size across all clusters (or, for an even number of clusters, the cluster with the smallest mean library size above the median). This uses the mean library size as a rough proxy for cell similarity. The baseline cluster is likely to be least dissimilar to every other cluster, which reduces the amount of DE during pairwise normalization between pseudo-cells. More intelligent choices of the baseline can be used if the similarities between clusters are known, e.g., from visualization after dimensionality reduction.

In general, cluster-specific normalization requires some caution. Done incorrectly, this may introduce artificial differences between cells in different clusters, such that the statistical rigour of downstream analyses (e.g., to detect DE between clusters) would be compromised. However, such problems are avoided here by using a two-step normalization strategy. The first normalization step removes any systematic differences between cells *within* each cluster, while the second normalization between pseudo-cells removes any differences *between* clusters. The end result is that differences between all cells in all clusters are removed. This is equivalent to the outcome of a hypothetical one-step method that does not use cluster information (and is robust to DE and stochastic zeroes, unlike existing methods). Moreover, normalization accuracy in Figure 4 is unaffected by the use of clustering prior to deconvolution, which suggests that this approach is valid.

Semi-systematic zeroes may be removed prior to deconvolution in each cluster. In particular, genes are removed if they only have zero counts across all cells in the cluster. Such genes provide no information for normalizing between cells in the same cluster, and their removal will not affect the cluster-specific size factor estimates. However, these genes are retained during rescaling of the size factors between clusters. This is because they will have non-zero counts in at least one cluster (assuming that systematic zeroes across the entire data set have already been removed). Removal of such genes will distort the median ratio between pseudo-cells and lead to biased size factor estimates, as described previously for the existing methods.

4 Assessing normalization in high-coverage simulations

The simulations in the main text represent low-coverage experiments similar to the Zeisel *et al.* and Klein *et al.* data sets. However, many scRNA-seq experiments involve higher sequencing coverage, e.g., over 2×10^5 reads per cell [12, 13], compared to approximately 2×10^4 in low-coverage experiments. To test the performance of deconvolution and the existing normalization methods in high-coverage scenarios, we repeat the simulations with some modifications. In particular, we redefine the sampling distribution of λ_{i0} such that

$$\log_2(\lambda_{i0}) \sim \text{Uniform}(3, 6)\ .$$

This provides a wider spread of abundances than in the original simulation, as the dynamic range of sequencing technologies increases with coverage. The NB dispersion is also re-defined for each gene as

$$\varphi_i = 2 + \frac{100}{\lambda_{i0}}$$

to represent a decreasing mean-dispersion trend. Note that the dispersions used here are larger than those in the low-coverage simulations. This is because zeroes are generally caused by dropouts during RNA capture. Increasing the coverage will only increase the counts in cells where the transcript is captured, while the counts in other cells remain at zero. Thus, as sequencing depth increases (and capture efficiency remains the same),

there will be a concomitant increase in the mean and variability of the counts. This is represented in the simulation by a larger dispersion value, especially at low abundances where dropouts are more likely.

The count for each gene in each cell was sampled from a NB distribution with a mean of $\theta_j \lambda_{is}$ and dispersion φ_i, using the same experimental design as described in the main text, i.e., three subpopulations of 250 cells with varying levels of DE. Size factors were then computed from the counts using the available methods. For the existing methods, similar performance was observed compared to the original simulations. DESeq and TMM normalization were consistently biased (Figures S4a and S4b), suggesting that zero counts are still problematic in high coverage data sets. Library size normalization continued to fail in the presence of DE genes (Figure S4c). In contrast, deconvolution performed well in all simulation scenarios (Figure S5). Greater divergence from the true size factors is observed in Figure S5c, but deconvolution is still more accurate than the existing methods in the equivalent scenario (Figure S4, third row). These results suggest that the increase in normalization accuracy from deconvolution is still present in higher-coverage experiments.

5 Computational complexity of the deconvolution method

The computational time and memory required by deconvolution depend on the number of cells. An equation is constructed for each cell, so the size of the linear system (in terms of the number of equations and coefficients) will increase quadratically with the number of cells. Furthermore, common implementations of the QR decomposition have $O(n^3)$ time complexity for n cells. This is roughly consistent with our empirical timings (Figure S6). In practice, the cubic time complexity is mitigated by the use of clustering to break up the linear system. As deconvolution is performed within each cluster, it is cubic only with respect to the size of each cluster, and is linear with respect to the number of clusters. The overall time complexity lies between the linear and cubic extremes, depending on the number of clusters and size of each cluster – a few large clusters will result in cubic complexity, while many small clusters will result in linear complexity.

Memory usage of the deconvolution method is harder to measure due to the unpredictability of garbage collection in R. We expect that usage will increase quadratically with the number of cells, due to increases to the size of the linear system. Again, this can be mitigated by clustering for large data sets. Further savings may be possible by using sparse matrices given that most of the entries in the system are equal to zero.

We note that most clustering algorithms have quadratic time complexity with respect to the number of cells, e.g., when building the distance matrix. However, we expect that clustering would be performed anyway as a routine part of the scRNA-seq data analysis. Thus, no additional time is added by normalization.

6 Comparing normalization accuracy on real data

6.1 Overview of the assessment framework

Consider two estimators Θ_j and Θ'_j of the size factor in cell j, where each estimator represents a different normalization method. We assume that Θ_j is an unbiased estimator of the true size factor θ_j, i.e., $E(\Theta_j) = \theta_j$. In contrast, Θ'_j is biased such that a power relationship exists between the estimates and true values. This is not unreasonable given the simulation results (Figure S4), and can be written as

$$\log[E(\Theta'_j)] = b \log(\theta_j)$$

for gradient b. Any bias will manifest as a non-unity gradient indicating divergence between the true and estimated values. (For simplicity, we do not include any intercept term in the above expression. This does not affect the generality of the framework, as the absolute scale of the size factors is irrelevant to their interpretation.) We define the true mean for gene i in cell j as $\mu_{ij} = \theta_j \lambda_{i0}$, where λ_{i0} represents the expected number of transcripts. We assume that no DE is present so a constant λ_{i0} can be used for all cells.

Now, consider fitting a log-link GLM to the counts using $\log[E(\Theta_j)]$ as an offset and $\log[E(\Theta'_j)]$ as the only covariate in a model with an intercept term. The systematic component of the GLM is written as

$$\log(\mu_{ij}) = \log[E(\Theta_j)] + \beta_{i0} + \log[E(\Theta'_j)]\beta_{i1}$$

where β_{i0} and β_{i1} are the values of the coefficients for the intercept and covariate terms, respectively. Given the relationships between $E(\Theta_j)$, $E(\Theta'_j)$ and θ_j, the above expression can be rewritten to obtain

$$\log(\theta_j) + \log(\lambda_{i0}) = \log(\theta_j) + \beta_{i0} + b\log(\theta_j)\beta_{i1}$$
$$\log(\lambda_{i0}) = \beta_{i0} + b\log(\theta_j)\beta_{i1} \ .$$

This relationship must hold for all cells as the systematic component of the GLM describes all contributions to μ_{ij} for all j. This means that, if $b \neq 0$, β_{i0} must be equal to $\log(\lambda_{i0})$ and β_{i1} must be equal to zero. We then perform a likelihood ratio test (LRT) against the null hypothesis of $\beta_{i1} = 0$ for the alternative hypothesis of $\beta_{i1} \neq 0$. We should obtain only a small number of rejections as the null hypothesis is true for each gene. (Similarly, if $b = 0$, few rejections should be observed as the null and alternative models are identical for an all-zero covariate.) In contrast, using $\log[E(\Theta'_j)]$ as the offset and $\log[E(\Theta_j)]$ as the covariate leads to

$$\log(\mu_{ij}) = \log[E(\Theta'_j)] + \beta'_{i0} + \log[E(\Theta_j)]\beta'_{i1}$$
$$\log(\theta_j) + \log(\lambda_{i0}) = b\log(\theta_j) + \beta'_{i0} + \log(\theta_j)\beta'_{i1}$$
$$\log(\lambda_{i0}) + (1-b)\log(\theta_j) = \beta'_{i0} + \log(\theta_j)\beta'_{i1} \ .$$

The above relationship only holds for all cells when $\beta'_{i0} = \log(\lambda_{i0})$ and $\beta'_{i1} = 1 - b$, where β'_{i0} and β'_{i1} are the values of the coefficients in this "switched" configuration. Repeating the LRT to test for a non-zero covariate term should yield more rejections than in the original configuration, as $\beta'_{i1} \neq 0$ when $b \neq 1$. These rejections can also be treated as DE genes where the DE log-fold change represents the normalization bias.

In practice, $E(\Theta_j)$ and $E(\Theta'_j)$ are unknown so we replace them with the observed size factor estimates $\hat{\theta}_j$ and $\hat{\theta}'_j$, respectively, in the GLM fit. This assumes that the variances of Θ_j and Θ'_j are low such that any observed estimate will be close to the expected value. The substitution is justified by the nature of the trends in Figures 1, 4, S4 and S5. For most scenarios, the estimates lie on a well-defined curve with little scatter which suggests that the estimation error is low. We also repeated the no-DE simulation in Figures 1 and 4 and computed the MAD of the log-estimates for each cell across 10 iterations. These MADs ranged from 0.007 to 0.021 for TMM normalization (using the interquartile range across all cells); 0.005 to 0.011 for DESeq normalization; 0.007 to 0.013 for library size normalization; and 0.025 to 0.043 for deconvolution. In contrast, the MAD of the log-transformed true size factors across cells was 0.39. This indicates that the estimation error of each size factor is small relative to the variability of the size factors across cells.

6.2 Deconvolution outperforms existing methods on real data

The above framework was applied to assess the performance of each existing method compared to the deconvolution method on real data. For each data set, cells were partitioned into pre-defined groups in which no DE was expected. The deconvolution size factors were log-transformed and used as offsets, while the log-size factors from an existing method were used as the covariates. NB dispersion estimation and GLM fitting was performed on the counts for each group separately, using methods from edgeR. The LRT was used to test for DE and the number of DE genes detected at a FDR of 5% was counted. This was repeated after switching the size factors, i.e., using the deconvolution size factors as the covariates and the existing size factors as the offsets. To ensure a valid comparison, the dispersion estimates from the original fit were re-used to fit the GLM after switching. Similarly, the p-value corresponding to an FDR threshold of 5% in the initial set of DE genes was used to define the set of DE genes in the switched fit. This means that the differences between β'_{i1} and β_{i1} are the sole cause of any difference in the number of DE genes. The entire procedure was then repeated for each existing method and for each group of cells. Examination of Figure S7 indicates that fewer DE genes are consistently detected in each group when the deconvolution size factors are used as the offsets, relative to the number detected when they are used as covariates. This suggests that the deconvolution size factors are equivalent to the unbiased estimates $\hat{\theta}_j$ and are more accurate than the existing methods.

For these results, there are a number of caveats that should be mentioned. Firstly, we assume one set of size factor estimates is unbiased, while the other set is not. The performance of deconvolution in the simulations suggests that it can be treated as the unbiased method. However, this is unlikely to be completely true as both sets will exhibit some bias. The above evaluation will be less effective when large biases are present in both the offsets and covariates. Secondly, we assume that the biased size factors follow a power relationship

with respect to the true values. The effect of other relationships is less predictable, though we would still expect them to yield non-zero values for β'_{i1}. Large $\text{var}(\Theta_j)$ and $\text{var}(\Theta'_j)$ may also increase the noise in the expected relationships of $\beta_{i1} = 0$ and $\beta'_{i1} = 1 - b$. Finally, we assume that there is no DE within the group of cells – or, at least, no DE that is associated with the size factors. We select cells in homogeneous groups to weaken this assumption, but there may be additional DE within each group that we have not considered. In summary, a large number of assumptions are used here, but this is likely to be inevitable given that the true size factors (which would facilitate a direct assessment of estimation accuracy) are unknown in real data.

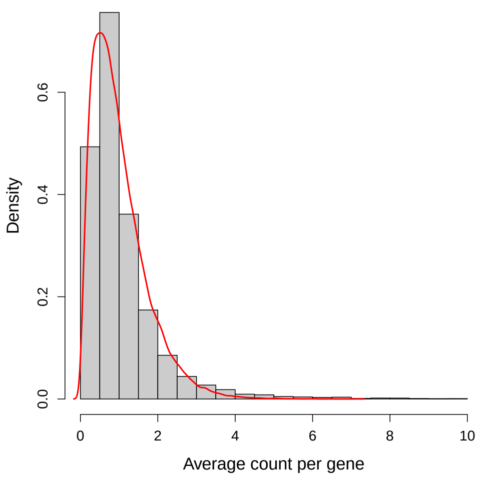

(a)

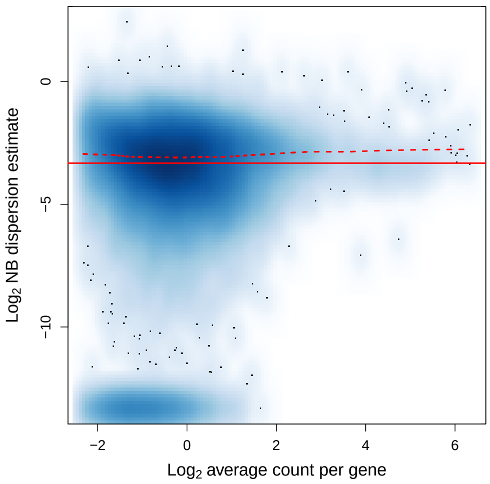

(b)

Figure S1: Parameter estimates for the Klein *et al.* data set [5]. (a) Histogram of average counts for all genes. The red curve represents the probability density of a Gamma$(2, 2)$ distribution. (b) Smoothed scatter plot of NB dispersion estimates, plotted against the average count. Dispersions were estimated using the estimateDisp function in edgeR, given a one-way layout with the before and after groups and using the log-transformed deconvolution size factors as the offsets. Axes are log-transformed for visibility. The dashed line represents the fitted trend from edgeR, while the unbroken line is drawn at the simulation value of 0.1.

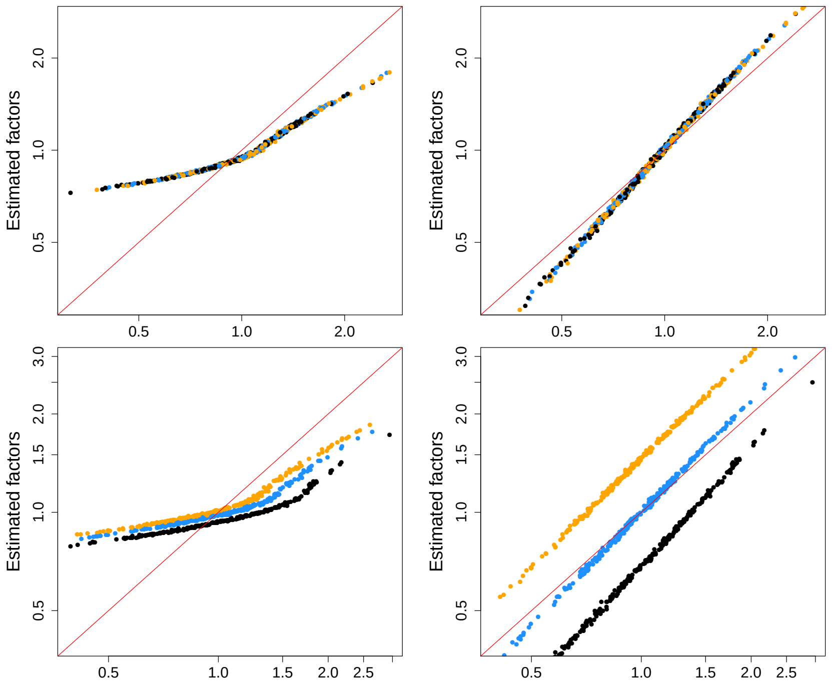

(a) **(b)**

Figure S2: Performance of DESeq normalization after addition of a pseudo-count. A pseudo-count of unity was added to (a) all counts directly, or (b) after scaling the pseudo-count by the relative library size, i.e., the pseudo-count added to each cell was equal to the ratio of its library size to the mean library size. Simulations were performed with no DE (first row) and varying magnitudes of DE (second row). Axes are shown on a log-scale. For comparison, each set of size factors was scaled such that the grand mean across cells was the same as that for the true values. The red line represents equality between the rescaled estimates and true factors. Cells in the first, second and third subpopulations are shown in black, blue and orange, respectively.

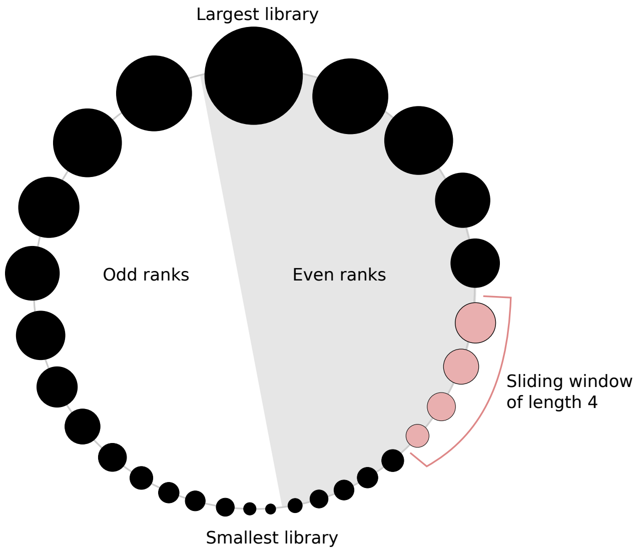

Figure S3: Ring arrangement of cells ordered by library size. Each circle represents a cell where the size of the circle corresponds to the library size of that cell. Even- and odd-ranking cells lie on opposite sides, with the largest and smallest libraries at the top and bottom, respectively. Cells lying in a window of length 4 are highlighted in red. Different instances of the window are obtained by sliding the window across the ring.

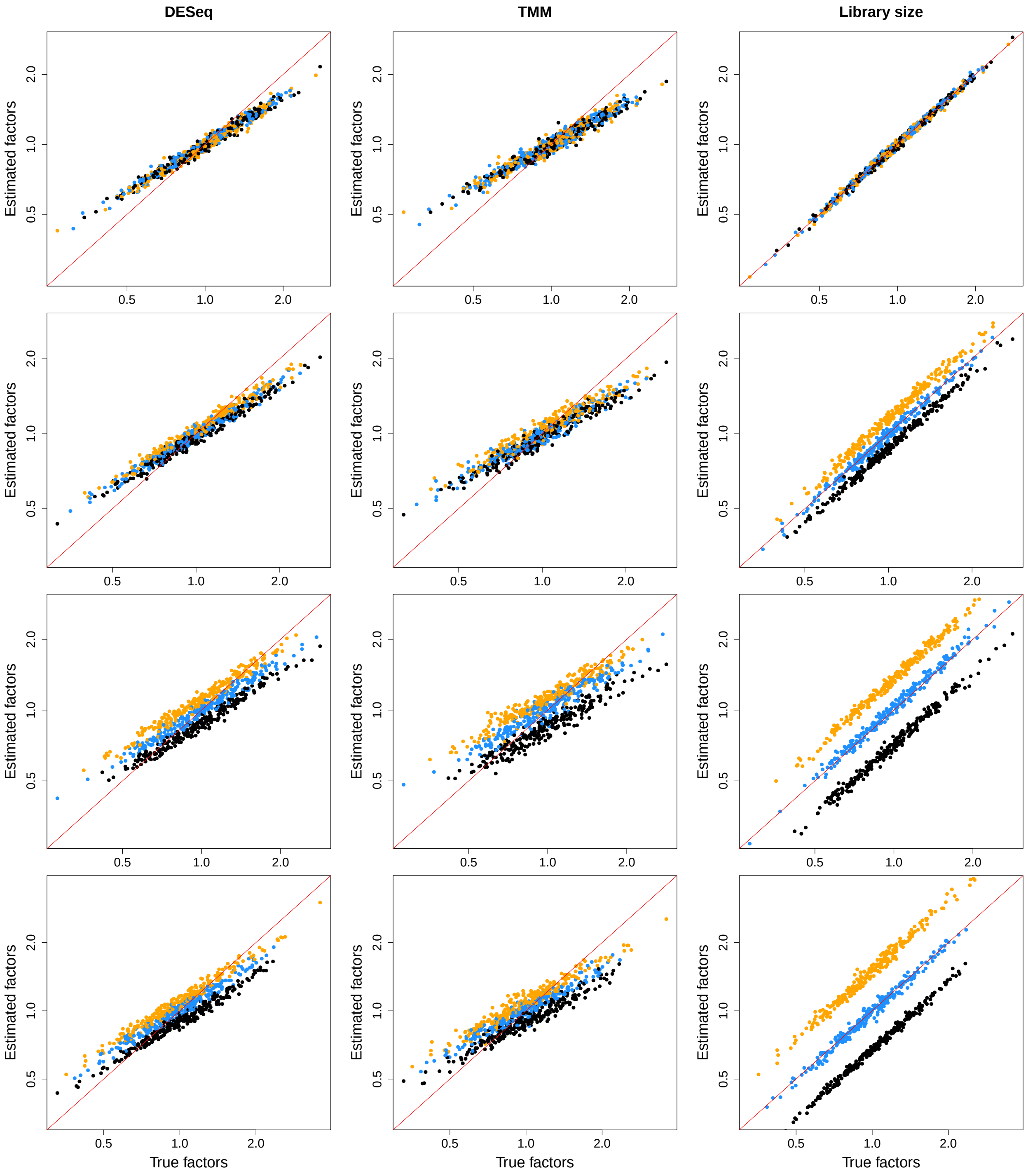

(a) **(b)** **(c)**

Figure S4: Performance of existing normalization methods in high-coverage simulations with DE genes and stochastic zeroes. The size factor estimates for all cells are plotted against the true values for (a) DESeq, (b) TMM and (c) library size normalization. Simulations were performed with no DE (first row), moderate DE (second row), strong DE (third row) and varying magnitudes of DE (fourth row). Axes are shown on a log-scale. For comparison, each set of size factors was scaled such that the grand mean across cells was the same as that for the true values. The red line represents equality between the rescaled estimates and true factors. Cells in the first, second and third subpopulations are shown in black, blue and orange, respectively.

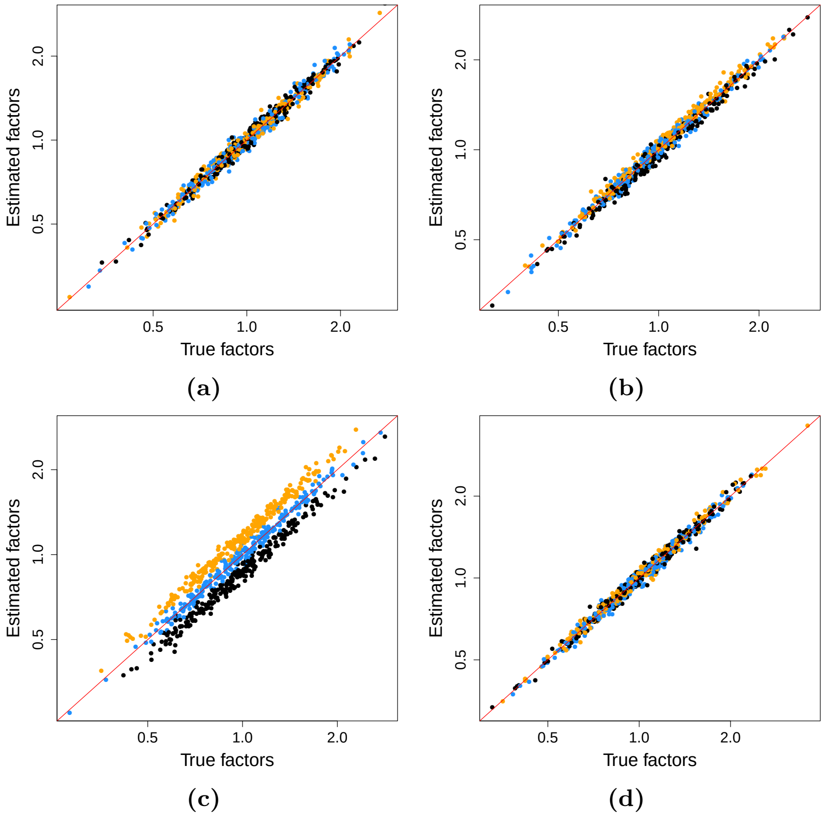

Figure S5: Size factor estimates from the deconvolution method in the high-coverage simulations, shown against the true values for scenarios with (a) no DE, (b) moderate DE, (c) strong DE and (d) varying magnitude of DE. Cells in the first, second and third subpopulations are shown in black, blue and orange, respectively. Axes are shown on a log-scale, and the red line represents equality with the true factors.

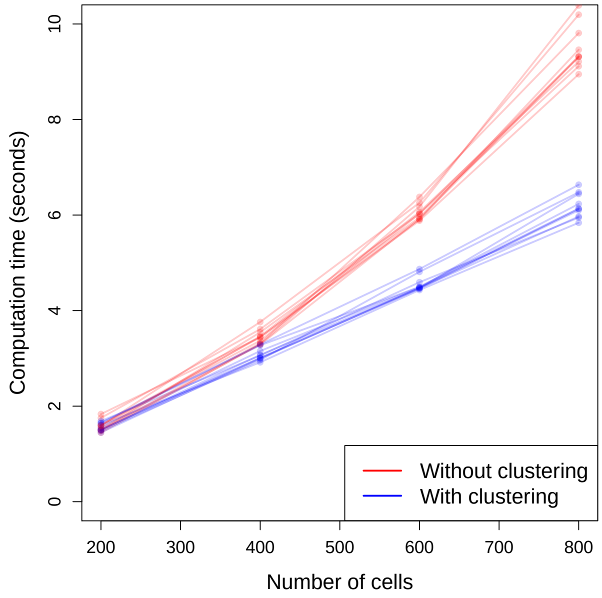

Figure S6: Computational time used by deconvolution on a simulated data set with varying numbers of cells. Counts were sampled from a Poisson distribution with a mean of 10, for 10000 genes across 200-800 cells. Deconvolution was applied with and without separation into clusters of 200 cells. This was repeated for 10 simulation iterations, where each line in the plot represents the timings for one iteration.

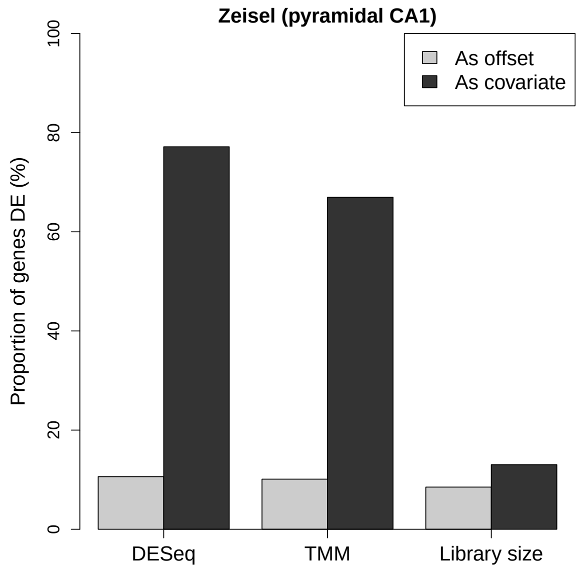

(a)

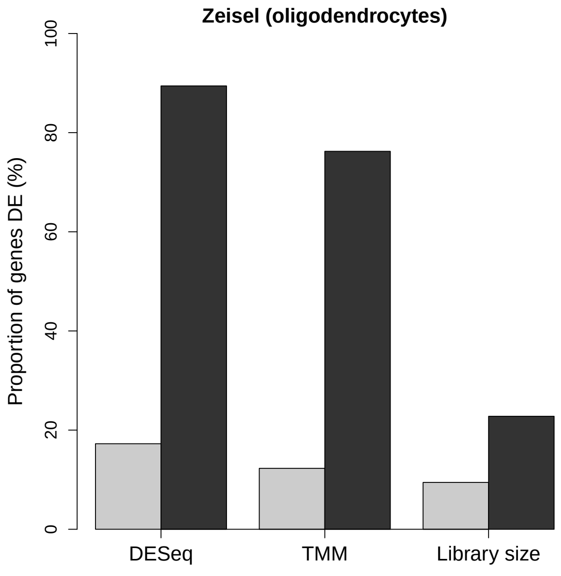

(b)

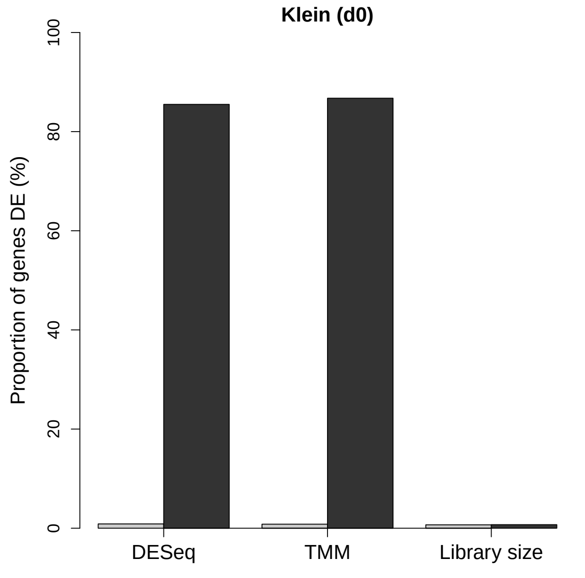

(c)

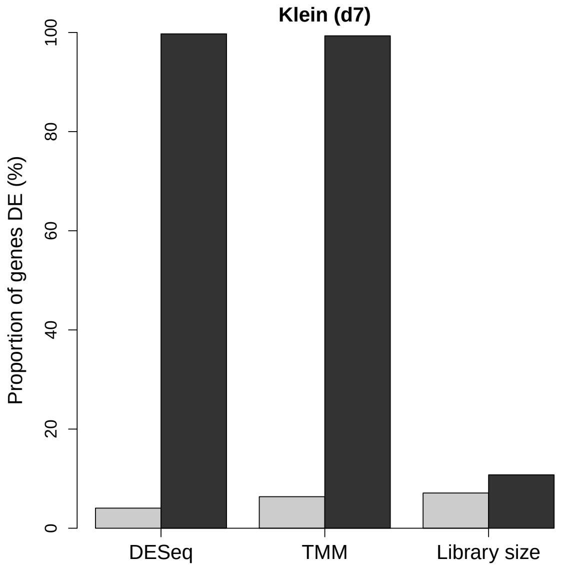

(d)

Figure S7: Number of DE genes detected by dropping the covariate term in the LRT, using either the deconvolution size factors as the offsets and size factors from each existing method as the covariates (light grey), or using the deconvolution size factors as the covariate and the existing size factors as the offsets (dark grey). Analyses were performed on the counts for (a) pyramidal CA1 cells or (b) oligodendrocytes in the Zeisel *et al.* data set [19], and (c) before or (d) after LIF withdrawal in the Klein *et al.* data set [5]. For the comparison between deconvolution and library size normalization, the significance of the difference in the proportion of DE genes between the offset and covariate analyses was quantified with Fisher's exact test, yielding p-values of (a) 2.4×10^{-22}, (b) 5.4×10^{-132}, (c) 0.77 and (d) 4.3×10^{-41}.

Table S1: Proportion of the top set of DE genes that were shared between deconvolution and each existing normalization method. Top genes were identified as those with the lowest p-values from edgeR in the analysis with each normalization method. Top sets of size ranging from 100 to 2000 were tested for both the brain and inDrop data sets. Smaller sets were not tested due to avoid ambiguous ranks from p-values of zero.

Data set	*Top*	*Method*		
		DESeq	TMM	Library size
Brain	100	0.44	0.60	0.71
	500	0.60	0.70	0.75
	2000	0.70	0.75	0.80
inDrop	100	0.88	0.94	0.98
	500	0.84	0.92	0.97
	2000	0.83	0.93	0.95

Table S2: Proportion of the top set of highly variable genes that were shared between deconvolution and each existing normalization method. Top genes were identified as those with the largest distance-to-median values. For both data sets, top sets of size ranging from 100 to 2000 were compared between methods.

Data set	*Top*	*Method*		
		DESeq	TMM	Library size
Brain	100	0.78	0.81	0.90
	500	0.78	0.80	0.88
	2000	0.67	0.73	0.84
inDrop	100	0.74	0.76	0.90
	500	0.57	0.59	0.85
	2000	0.59	0.61	0.85
